# Gastric Cancer-Derived Extracellular Vesicles (EVs) Promote Angiogenesis via Angiopoietin-2

**DOI:** 10.3390/cancers14122953

**Published:** 2022-06-15

**Authors:** Talya Kalfon, Shelly Loewenstein, Fabian Gerstenhaber, Stav Leibou, Hen Geller, Osnat Sher, Eran Nizri, Guy Lahat

**Affiliations:** 1 Division of Surgery, Tel Aviv Sourasky Medical Center, Tel Aviv 6423906, Israel; talyachlfon@mail.tau.ac.il (T.K.); fabiang@tlvmc.gov.il (F.G.); stavleibou@mail.tau.ac.il (S.L.); hengeler@mail.tau.ac.il (H.G.); erann@tlvmc.gov.il (E.N.); guyla@tlvmc.gov.il (G.L.); 2 Institute of Pathology, Tel-Aviv Sourasky Medical Center, Tel-Aviv 6423906, Israel; osnats@tlvmc.gov.il; 3 The Sackler Faculty of Medicine, Tel Aviv University, Tel-Aviv 6997801, Israel

**Keywords:** gastric cancer (GC), endothelial cells (ECs), angiogenesis, angiopoetin-2 (ANG2), extracellular vesicles (EVs)

## Abstract

**Simple Summary:**

Angiogenesis is the formation of new blood vessels, which is essential for gastric cancer growth and metastasis. Angiopoietin-2 is a key driver of tumor angiogenesis and has recently emerged as a promising target for antiangiogenic therapy. Extracellular vesicles play an important role in tumor progression including angiogenesis. We explored the crosstalk between gastric cancer and endothelial cells mediated by vesicles, with a specific focus on angiopoietin-2. We show that primary gastric cancer and omental metastasis tissues express angiopoietin-2. We isolated gastric cancer vesicles and demonstrated that they induce the proliferation, migration, invasion, and tube formation of endothelial cells. Characterization of the angiogenic profile of these vesicles revealed high levels of proangiogenic proteins including angiopoietin-2. Using angiopoietin-2 knockdown, we demonstrate that angiopoietin-2 mediates the proangiogenic effects of the gastric cancer vesicles. Our findings suggest a new mechanism via which gastric cancer cells induce angiogenesis. Such a mechanism may be used as a target for cancer therapy.

**Abstract:**

Angiogenesis is an important control point of gastric cancer (GC) progression and metastasis. Angiopoietin-2 (ANG2) is a key driver of tumor angiogenesis and metastasis, and it has been identified in primary GC tissues. Extracellular vesicles (EVs) play an important role in mediating intercellular communication through the transfer of proteins between cells. However, the expression of ANG2 in GC-EVs has never been reported. Here, we characterized the EV-mediated crosstalk between GC and endothelial cells (ECs), with particular focus on the role of ANG2. We first demonstrate that ANG2 is expressed in GC primary and metastatic tissues. We then isolated EVs from two different GC cell lines and showed that these EVs enhance EC proliferation, migration, invasion, and tube formation in vitro and in vivo. Using an angiogenesis protein array, we showed that GC-EVs contain high levels of proangiogenic proteins, including ANG2. Lastly, using Lenti viral ANG2-shRNA, we demonstrated that the proangiogenic effects of the GC-EVs were mediated by ANG2 through the activation of the PI3K/Akt signal transduction pathway. Our data suggest a new mechanism via which GC cells induce angiogenesis. This knowledge may be utilized to develop new therapies in gastric cancer.

## 1. Introduction

Gastric cancer (GC) is the third leading cause of cancer mortality worldwide [1]. Despite considerable improvements in surgical quality and multidisciplinary treatment, this high mortality rate is mainly a result of late diagnosis and limited treatment options for recurrent and metastatic disease [2]. Nevertheless, in the past decade, novel therapies were explored and implemented in clinical practice. These, include biological agents and immunotherapies which target the tumor and its microenvironment [3,4,5].

Angiogenesis, defined as the rapid formation of novel blood vessels and increased vascular permeability of existing ones, is one of the most critical steps in the development and metastasis of solid tumors. Endothelial cells (ECs), which line the interior surface of blood vessels, are the central component in this process. Under specific biochemical conditions, ECs cause the degradation of the vascular basement membrane, proliferate, and migrate into the perivascular stroma, leading to the formation of tubular structures and tissue neovascularization [6,7]. During tumorigenesis, the balance between proangiogenic and antiangiogenic molecules leans towards proangiogenic molecules, promoting the expression of various angiogenic factors by both tumor and stromal cells [8,9]. Angiopoietins 1–4 (ANG1–4) are a family of secreted factors that play a central role in the angiogenic switch during tumor progression. The most comprehensively studied angiopoietins are ANG1 and ANG2. While ANG1 has a critical role in physiological angiogenesis [10], ANG2 is more dominant in inflammatory and cancer-related angiogenesis, usually through activation of the phosphoinositide3-kinase (PI3K)/Akt pathway via the TIE2 receptor [11,12,13]. ANG2 is expressed in gastric cancer tissues by both cancer cells and endothelial cells [14]. Moreover, its level of expression directly correlates with tumor stage [15], suggesting that the ANG–TIE pathway has a potential role in gastric cancer spread and its adverse clinical outcomes [16,17,18].

Extracellular vesicles (EVs) are small nano spherical particles with an outer lipid bilayer, which are secreted from cells into the extracellular space and are involved in the communication between cells [19]. EVs are classified into different subtypes according to their size, biogenesis, content, and function. They include larger EVs, such as microvesicles (100–1000 nm) that are shed from the cell membrane, and smaller EVs, which include exosomes (50–150 nm) secreted from endocytic pathways [20,21,22]. The complex network of communication between cancer cells and their microenvironment mediated by EVs is critical in the various stages of cancer progression due to their regulatory impact on immunity, angiogenesis, proliferation, invasion, and metastasis [23,24,25]. These effects are mediated by the content of EVs, including RNA (mRNA, miRNA, and other RNAs), proteins, and lipids [26,27]. While knowledge on the role of tumor-derived EVs in angiogenesis is expanding [28,29], research on the role of GC-derived EVs (GC-EVs) in angiogenesis is still in its early stages and mainly focuses on EVs-derived miRNA [30,31]. We characterized the angiogenic protein content of GC-EVs, demonstrating their proangiogenic in vivo and in vitro effects, specifically through ANG2.

## 2. Materials and Methods

### 2.1. Cell Culture

AGS and SNU-16 human gastric adenocarcinoma cells and EA.hy926 human endothelial cells were purchased from the American Tissue Culture Collection (ATCC) (Biological Industries Ltd., Beit Haemek, Israel). Cells were identified as being *Mycoplasma*-free by a PCR-based method (Hy-mycoplasma Detection Kit, Hylabs, Israel) and cultured for no more than 20 passages between thawing and use in experiments. AGS and EA.hy926 cells were cultured in Dulbecco’s modified Eagle’s medium (DMEM), and the SNU-16 cells (grown in suspension) were cultured in RPMI-1640 medium. Both were supplemented with 10% heat-inactivated fetal bovine serum (FBS) and 100 U/mL penicillin-streptomycin (Biological Industries Ltd., Beit Haemek, Israel). The cells were maintained in a humidified 5% CO_2_ atmosphere at 37 °C. All cells treated with EVs were grown in medium supplemented with 10% EV-depleted FBS prior to the assay.

### 2.2. EV Isolation from AGS and SNU-16 Cell Lines

Gastric cancer small EVs (GC-EVs), enriched in exosomes, were obtained by differential centrifugation from culture medium conditioned by AGS and SNU-16 cells grown in 200 mL of serum free medium for 24 h, as we previously described [32]. Briefly, conditioned medium was centrifuged at 3000× *g* for 10 min to remove cell debris and most “large” EVs. The resulting supernatant was further centrifuged for 30 min at 10,000× *g* to remove most “medium” EVs; finally, small EVs were enriched by ultracentrifugation at 100,000× *g* for 70 min. The small EV pellet was suspended in PBS and recentrifuged at 100,000× *g* for 70 min [33]. All steps were carried out at 4 °C. Using this protocol, EV characterization revealed the presence of particles with morphological and biochemical characteristics of small EVs, in accordance with the guidelines of the Minimal Information for Studies of Extracellular Vesicles (MISEV 2018) [34]. The pelleted exosomes were resuspended in PBS and quantified using the Bradford assay (Bio-Rad, Hercules, CA, USA).

### 2.3. Cryogenic Transmission Electron Microscopy (Cryo-TEM)

Specimens were prepared and analyzed on a Thermo-Fisher Talos F200C, FEG-equipped high resolution-TEM, operated at 200 kV as previously described [35,36,37].

### 2.4. Nanoparticle Tracking Analysis (NTA)

The analysis of size distribution of EVs based on Brownian motion was assayed by NanoSight NS300 (NanoSight, Amesbury, UK) as previously described [32]. Briefly, the EV fraction was diluted 1:1000 with 0.1 µm filtered PBS, and size dispersion was measured at 25 °C. At least three videos of 30 s in duration were taken with a frame rate of 30 frames/second, and particle movement was analyzed by NTA software (version 3.1, NanoSight, Salisbury, UK).

### 2.5. Western Blot Analysis

Cells and EVs were lysed in RIPA buffer (Merck). Proteins (20 μg for cells and 6 µg for EVs) were electrophoresed on SDS-PAGE. Western blotting analyses were performed using the following antibodies: mouse monoclonal anti-human CD81 (Santa-Cruz Biotechnology; SC-166029), mouse monoclonal anti-human CD63 (Santa-Cruz Biotechnology; SC-5275), rabbit monoclonal anti-human CD9 (abcam; ab92726), rabbit monoclonal anti-human Ang2 (abcam; ab155106), rabbit polyclonal anti-human p-Akt (Cell Signaling technology; #9271), rabbit polyclonal anti-human p-ERK1/2 (Cell Signaling technology; #9101), mouse monoclonal anti-human PI3K (abcam; ab86714), and mouse monoclonal anti-human beta-Actin (Cell Signaling technology; #3700). For the expression of ANG2, p-Akt, p-ERK1/2 and PI3K, the cells were cocultured with 10 µg/mL EVs for 24 h prior to analysis. Densitometry readings were measured using FUSION FX software and were normalized to the corresponding β-actin. The original WB can be found in Figures S1 and S2.

### 2.6. EVs Labeling

EVs labeling was performed as described by Hazan-Halevy et al. and by our group [32,38].

### 2.7. EVs Internalization Assay

Internalization was measured by confocal microscopy and flow cytometry analysis as previously described [32] using EA.hy926 cells (3 × 10^5^ and PKH-67-labeled-EVs (2 μg). Data were acquired by FACS Canto II with Diva software (Becton Dickinson, Franklin Lakes, NJ, USA) and analyzed using FlowJo software (Tree Star, Inc., Ashland, OR, USA). For confocal microscopy analysis, the slides were viewed under a Zeiss LSM700 confocal microscope.

### 2.8. Cell Growth Assay

Cell proliferation was measured using the XTT cell proliferation kit (Biological Industries) according to the manufacturer’s instructions and as described previously [32] using 5000 EA.hy926 cells/well and 10 µg/mL EVs for 24 h.

### 2.9. Migration and Invasion Assays

Transwell migration and invasion assays were conducted as described previously [32] using 5 × 10^4^ EA.hy926 cells suspended in serum-free medium (0.5 mL/chamber) in the upper chamber of 24-well Transwell inserts with 8 mm pore size (BD Biosciences). The lower chamber was filled with 0.75 mL/well DMEM with 2% EV-depleted FBS and 10 μg/mL EVs. In all cases, the cells were incubated for 16 h at 37 °C. The migratory and invasive activities were determined by counting the number of cells in three fields per well (magnification, 100×) in triplicate with the ImageJ 1.48v.Java image processing program.

### 2.10. Tube Formation Assay

In vitro angiogenesis was measured using an endothelial tube formation assay. Briefly, 1 × 10^5^ EA.hy926-GFP, shCtrl EA.hy926-GFP or shANG2 EA.hy926-GFP cells were plated on a Matrigel (BD Biosciences)-coated 96-well plate in no serum (EA.hy926-GFP) or 10% FBS (shCtrl EA.hy926-GFP and shANG2 EA.hy926-GFP) with the addition of 10 μg/mL shCtrl-AGS-EVs, shANG2-AGS-EVs, 200 ng/mL human recombinant ANG2 (R&D systems), or 5µM TIE2 inhibitor (abcam, ab141270-B). After 12 to 18 h, the plates were examined under a fluorescence microscope for the formation of tubes and photographed. The number of tubes in the treatment and control groups was measured in five random fields (magnification, 100×).

### 2.11. Matrigel Plug Assay

All animal procedures and care were approved by the Institutional Animal Care and Usage Committee of the Tel Aviv Sourasky Medical Center (protocol # 24-8-19). Six week old male athymic nude mice Foxn1^nu/+^ were injected subcutaneously with 500 μL of Matrigel (BD Biosciences) containing 100 μg AGS-EVs or PBS as a control. The mice were sacrificed after 14 days, and the Matrigel plugs were surgically removed and analyzed for hemoglobin content with Drabkin’s reagent kit (Sigma-Aldrich) and for the expression of CD31 using immunohistochemistry (IHC) [39].

### 2.12. Immunohistochemistry (IHC)

Immunohistochemistry was performed on formalin-fixed, paraffin-embedded tissue sections of 4 µM as described elsewhere [40]. The staining itself consisted of primary antibodies for ANG2 (Santa-Cruz Biotechnology; sc-74403) and CD31 (abcam; ab28364). Staining with pan-cytokeratin (Novus; AE-1/AE-3) was performed with the Ventana Benchmark automated staining system (Ventana Medical Systems, Tucson, AZ) on 4 μm paraffin sections as previously described [32]. The intensity of ANG2 staining was scored by a gastrointestinal pathologist (O.S.) as none (=0), weak (=1), moderate (=2), or strong (=3).

### 2.13. Human Angiogenesis Antibody Array

The content of EV angiogenesis-related proteins was screened by the Human Angiogenesis Antibody Array G1000 (RayBiotech, Peachtree, GA, USA) according to the manufactures’ instructions using 100 µg of total protein obtained from AGS-EVs. Fluorescence intensity was analyzed using a microarray scanner (Innopsys InnoScan^®^, Chicago, IL, USA) and its software, which calculated the mean intensity of each spot minus background and the negative control. The results were normalized to positive control spots.

### 2.14. Lentiviral Knockdown of ANG2

Mission shRNA plasmid DNA pLKO.1-puro-shANG2 clones 3 and pLKO.1-puro-shControl (Sigma Aldrich) were transfected along with pCMV-VSV-G and psPax2 (1:1:1 µg) into HEK-293T cells using lipofectamine 2000 (Invitrogen). In brief, HEK-293T cells were seeded in six-well plates and grown to 80% confluence. Plasmid DNA (3 µg) was mixed with 7 µL of Lipofectamine 2000 reagent in 250 µL of Opti-MEM and incubated for 20 min at room temperature. The mixed solution was added to cells for 24 h. Conditioned medium containing viral particles was harvested and filtered through 0.45 mm filters. For virus infection, EAhy.926 or AGS cells were incubated with conditioned media containing virus particles supplemented with polybrene (8 µg/mL) for 8 h. Stably infected cells were selected by puromycin (0.5 µg/mL). ANG2 knockdown was confirmed using qRT-PCR and Western blot analysis.

### 2.15. Statistical Analysis

Statistical analysis was performed using GraphPad Prism^TM^ software. Continuous variables were compared using the *t*-test for significance. All significance tests were two-tailed, and a *p*-value of ≤0.05 was considered statistically significant. Results are presented as the mean ± standard deviation (SD).

## 3. Results

### 3.1. ANG2 Is Expressed in Human Gastric Cancer and Omental Metastasis Samples

**Figure 1:**
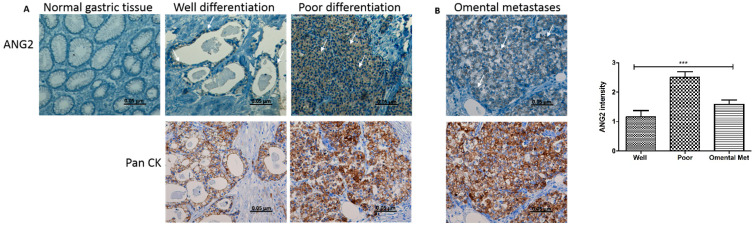
Expression of ANG2 in human gastric cancer tissues. (**A**) IHC staining of ANG2 and Pan CK in matched normal gastric tissue and primary GC (*n* = 12). (**B**) Expression of ANG2 and Pan CK in GC omental metastatic tissues (*n* = 12); arrowhead, ANG2-positive cells. Representative images are shown (magnification, 200×; scale bar represents 0.05 µm). IHC quantification is shown on the right. *** *p* < 0.001.

Initially, the slides were stained for pan-cytokeratin (pan CK) to confirm the presence of tumor and metastatic cells in the evaluated tissues (Figure 1A,B) [41]. Primary and metastatic cells demonstrated a high expression level of pan-cytokeratin. To evaluate the clinical relevance of angiopoietins in gastric cancer (GC), we assessed the expression levels of ANG2 in tissues of primary gastric tumors and in matched normal gastric tissues (*n* = 12 each). As depicted in Figure 1A, IHC staining for ANG2 showed higher expression levels in tumor cells of primary gastric tumors compared to normal gastric tissues and revealed a trend toward a positive correlation between poor tumor differentiation and the level of expression. We then stained matched specimens of GC omental metastases, a common site for GC spread (*n* = 12). As shown in Figure 1B, we observed moderate expression of ANG2 in the metastatic tumoral tissues. These data support the premise that angiopoietins have a potential role in GC progression and metastasis, most probably via induction of angiogenesis. We next sought to investigate whether GC-induced angiogenesis is mediated by angiopoietins secreted and delivered by GC-EVs.

### 3.2. Isolation and Characterization of Gastric Cancer-Derived EVs

**Figure 2:**
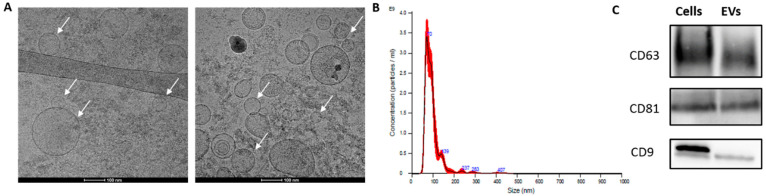
Characterization of gastric cancer-derived EVs. (**A**) Analysis of AGS-EVs by cryogenic transmission electron microscopy (cryo-TEM); arrowhead, a gastric cancer-derived extracellular vesicle. Scale bar represents 100 nm. (**B**) Measurement of AGS-EVs by nanoparticle tracking analysis system. (**C**) Western blot analysis of the EV markers CD63, CD81, and CD9. The original WB can be found in Figure S1.

To investigate the effect of GC-EVs on angiogenesis, we first purified and characterized small EVs from the conditioned medium (CM) of the human gastric cancer cell line AGS. Isolation was performed using differential ultracentrifugation according to the current gold standard for EV isolation and as we have previously described [32,33]. EV count, size, markers, and morphology were characterized by nanoparticle tracking, Western blot, and cryo-TEM in accordance with the Minimal Information for Studies of Extracellular Vesicles (MISEV 2014 and 2018) guidelines [32,34,42]. As can be seen in Figure 2A, the cryo-TEM images revealed the presence of round particles with dimensions of 65–200 nm. Nanoparticle tracking analysis (NTA) demonstrated a homogeneous population of EVs with a modal diameter of 68.9 ± 1.0 nm, which is within the range of small EVs (sEVs, <100 nm) [34], and a concentration of 1.68 × 10^11^ ± 2.32 × 10^10^ particles/mL (Figure 2B). The EV preparations also contained 10% large EVs with a diameter >134.3 nm as analyzed by NTA (D90 = 134.3 ± 3.0 nm). As depicted in Figure 2C, CD63, CD81, and CD9 were highly expressed in both AGS cells and AGS-derived EVs. These results confirmed the secretion of EVs from GC cells.

### 3.3. Uptake of Gastric Cancer Derived EVs by Endothelial Cells

**Figure 3:**
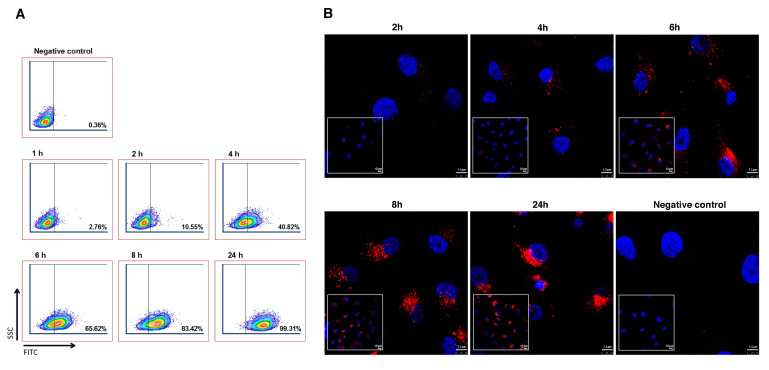
Uptake of gastric cancer-derived EVs by endothelial cells. (**A**) Internalization of PKH-67-labeled AGS-EVs measured by flow cytometry. (**B**) Internalization of PKH-26-labeled AGS-EVs measured by confocal analysis. Negative control, AGS cells with no addition of labeled EVs. Scale bar represents 7.5 µm and 10 µm.

EVs must be taken up by their target cells in order to assess protein delivery via EVs. For that purpose, we incubated fluorescent dye-labeled AGS-EVs with EAhy.926 transformed human umbilical vein endothelial cells (HUVECs) at different timepoints and followed their uptake by quantitative flow cytometry (Figure 3A) and confocal microscopy analysis (Figure 3B). We observed EV uptake by endothelial cells as early as 1 h following incubation (2.76%). Longer incubation times resulted in greater accumulation of EVs inside the cells. As depicted in Figure 3A, 10.55% of EVs were taken up by the endothelial cells after 2 h of incubation, rising to 83.42% after 8 h of incubation. These results confirmed the rapid uptake of gastric cancer-derived EVs by endothelial cells (ECs).

### 3.4. Gastric Cancer-Derived EVs Promote Proliferation, Migration, and Invasion of Endothelial Cells

**Figure 4:**
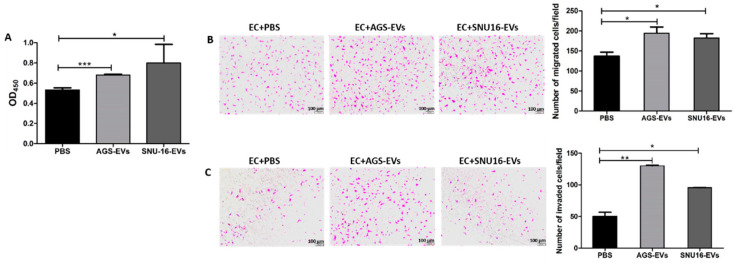
Gastric cancer-derived EVs promote the proliferation, migration, and invasion of endothelial cells. (**A**) AGS-EVs and SNU-16-EVs promote endothelial cell (EC) proliferation. The data are presented as the mean ± SD of three independent experiments. (**B**) AGS-EVs and SNU-16-EVs promote endothelial cell migration and (**C**) invasion. The left panel depicts representative images (magnification, 100×; scale bar represents 100 µm), and the right panel graphs represent the average of three repeated experiments ± SD. * *p* < 0.05; ** *p* < 0.01; *** *p* < 0.001.

Next, we examined whether uptake of GC-EVs induces proangiogenic properties in endothelial cells. For that purpose, we used EVs isolated from two different gastric cancer cell lines: AGS and SNU-16. AGS-EVs and SNU-16-EVs were cocultured with EAhy.926 endothelial cells (ECs), and their effect on proliferation was detected using an XTT cell proliferation kit. Our results demonstrated that both AGS-EVs and SNU-16-EVs increased endothelial cellular proliferation by 30% (*p* < 0.001) and 50% (*p* = 0.0288), respectively (Figure 4A). Similarly, AGS-EVs and SNU-16-EVs increased migration and invasion of endothelial cells in a Boyden chamber assay, migration by 1.5-fold (*p* = 0.0352) and 1.3-fold (*p* = 0.0328), respectively, and invasion by 2.6-fold (*p* = 0.0027) and 1.9-fold (*p* = 0.0132), respectively (Figure 4B,C). Taken together, these results demonstrated that GC-EVs induce phenotypic changes in ECs favoring angiogenesis.

### 3.5. Gastric Cancer-Derived EVs Promote Tube Formation In Vitro and Blood Vessels In Vivo

**Figure 5:**
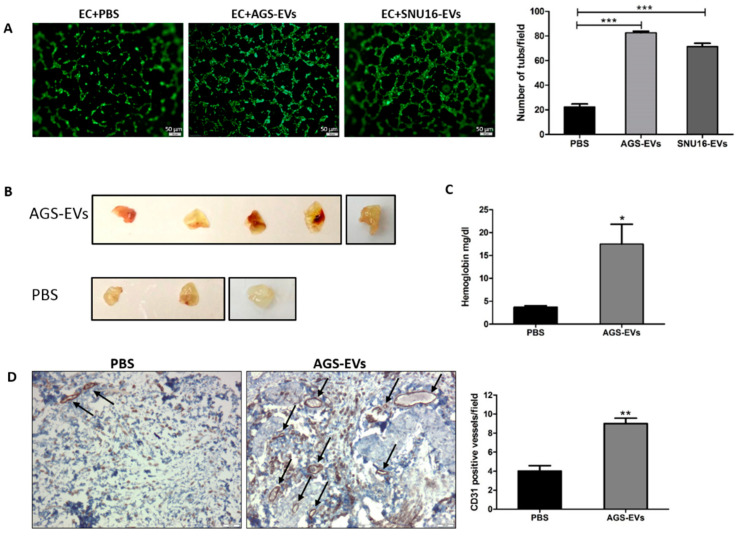
Gastric cancer-derived EVs promote tube formation in vitro and blood vessels in vivo. (**A**) AGS-EVs and SNU-16-EVs promote tube formation of endothelial cells (ECs) in vitro. The left panel depicts representative images (magnification, 100×; scale bar represents 50 µm), and the right panel graphs represent the average of three repeated experiments ± SD. (**B**) AGS-EVs promote blood vessel formation in vivo (*n* = 5 for AGS-EVs and *n* = 3 for PBS). Images depict surgically removed Matrigel plugs after 14 days. (**C**) Quantification of hemoglobin content in Matrigel plugs using Drabkin reagent. (**D**) CD31 IHC staining of blood vessels in Matrigel plugs; arrowhead, CD31-positive cells. Representative images are shown on the left (magnification, 100×; scale bar represents 100 µm). IHC quantification is shown on the right. * *p* < 0.05; ** *p* < 0.01; *** *p* < 0.001.

We next performed an in vitro endothelial tube formation assay. Both AGS-EVs and SNU-16-EVs increased the formation of endothelial tubes by 3.7-fold (*p* < 0.001) and 3.2-fold (*p* < 0.001), respectively (Figure 5A). We then performed an in vivo Matrigel plug assay in which Matrigel mixed with PBS (control) or AGS-EVs was injected subcutaneously into nude mice. As shown in Figure 5B, AGS-EVs significantly increased vascularization within the Matrigel plugs. Figure 5C depicts a significant increase in the hemoglobin content within the Matrigel plugs injected with AGS-EVs: 17.5 mg/dL in the AGS-EVs vs. 3.8 mg/dL in the control-treated mice (*p* = 0.046). In addition, CD31 immunohistochemical (IHC) staining of sections from the Matrigel plugs demonstrated a significant increment in the number of blood vessels: nine CD31-positive vessels/field in the AGS-EVs vs. four in the control-treated mice (*p* = 0.0036) (Figure 5D). Taken together, these data suggest that GC-EVs promote angiogenesis both in vitro and in vivo.

### 3.6. Gastric Cancer-Derived EVs Contain Various Angiogenic Proteins

**Figure 6:**
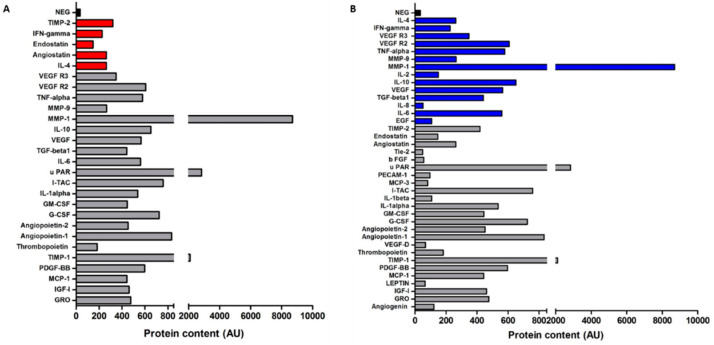
Gastric cancer-derived EVs contain various angiogenic proteins. (**A**) The red bars represent antiangiogenic proteins. (**B**) The blue bars represent previously described GC-EV-derived proteins. The graphs display signal intensity of each protein. Proteins above 100 AU are presented.

To identify potential proteins within AGS-EVs that may be responsible for the observed proangiogenic effects on endothelial cells, we characterized its protein content using a human angiogenesis protein antibody array which detects 43 different angiogenic proteins. As depicted in Figure 6A, the analysis revealed high levels of various angiogenic proteins. Most of these factors have been reported as proangiogenic e.g., angiopoietin-1 (ANG1), angiopoietin-2 (ANG2), VEGF, MMP1, uPAR, I-TAC (CXCL11), IL-6, IGF-I, and TIMP-1 (protein content (AU): 833, 455, 566, 8701, 2836, 758, 561, 461, and 2100, respectively; Figure 6A), while others as antiangiogenic, e.g., angiostatin, endostatin, IL-4, TIMP-2, and INF-gamma (protein content (AU): 263, 147, 263, and 226, respectively; Figure 6A) [28]. The expression levels of most proangiogenic proteins (approximately 64%) were higher than those of the antiangiogenic factors. Moreover, according to the exosome and extracellular vesicles database ExoCarta and EVpedia [43,44], 14 proteins (33%) were previously found in GC-EVs (Figure 6B). In contrast, ANG1 and ANG2, as well as several other proangiogenic proteins, have not been previously reported in relation to GC-EVs. In support of our hypothesis and previous findings, these data demonstrate that GC-EVs contain numerous proangiogenic proteins, including ANG1 and ANG2.

### 3.7. Gastric Cancer-Derived EVs Increase the Expression of ANG2 in Endothelial Cells

**Figure 7:**
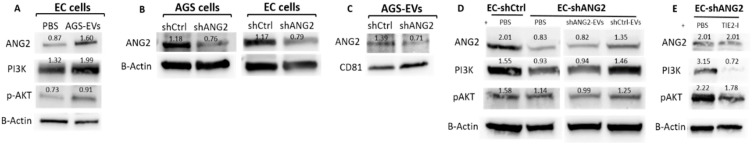
Gastric cancer-derived EVs increase the expression of ANG2 in endothelial cells. (**A**) The expression of ANG2 was increased in endothelial cells (ECs) treated with AGS-EVs. (**B**) The expression of ANG2 was decreased in ANG2 knockdown AGS cells and ECs (shANG2) compared to control cells (shCtrl). (**C**) AGS-EVs isolated from shANG-AGS cells contained reduced levels of ANG2 compared to EVs isolated from shCtrl-AGS cells. (**D**) AGS-EVs induced the expression of ANG2 and its signal transduction pathway in EA.hy926 ECs. (**E**) Treatment of ECs with TIE2 inhibitor (TIE2-I) reduced the expression of PI3K and p-Akt in ECs. The original WB can be found in Figure S2.

We first examined the effect of AGS-EVs on the expression of ANG2 and two of its downstream proteins: PI3K and p-Akt, in naïve endothelial cells. As depicted in Figure 7A, there was an increase in the expression of ANG2, PI3K, and p-Akt in these AGS-EV-treated endothelial cells. Next, in order to investigate the specific role of GC-EV-derived ANG2, we created an in vitro model of ANG2 knockdown GC and endothelial cells (ECs). Figure 7B demonstrates a successful ANG2 knockdown in both cell lines. Consequently, lower expression levels of ANG2 were also observed in EVs from knockdown GC cells (shANG2-EVs) compared to the control EVs (shCtrl-EVs; Figure 7C). Next, we treated ANG2 knockdown ECs with shANG2-EVs vs. shCtrl-EVs and evaluated the expression levels of ANG2 and two of its downstream proteins, PI3K and p-Akt, in these endothelial cells. As shown in Figure 7D, there was a significantly higher expression of ANG2, PI3K, and p-Akt in the endothelial cells that were treated with the ANG2-positive EVs (shCtrl-EVs). Lastly, an inhibitor of TIE2 was used in order to specifically assess the involvement of the PI3K/Akt pathway. As can be seen in Figure 7E, treatment of the shANG2-ECs with the TIE2 inhibitor (TIE2-I) reduced the expression of both PI3K and p-Akt. Taken together, these results suggest that GC-EVs transfer ANG2 to endothelial cells, thus activating the PI3K/Akt signal transduction pathway.

### 3.8. ANG2 Derived from Gastric Cancer EVs Mediates Gastric Cancer Induced Angiogenesis

**Figure 8:**
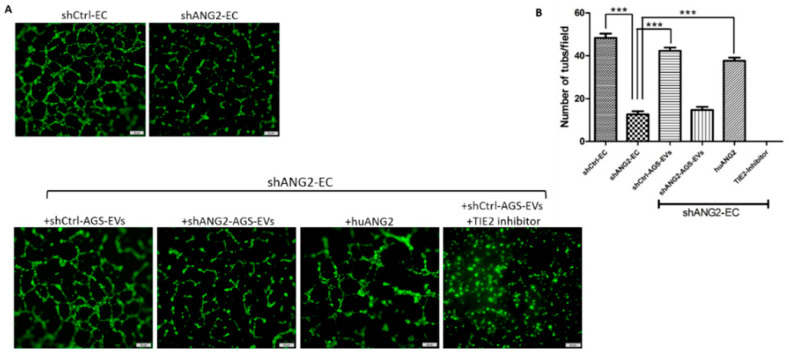
ANG2 derived from GC-EVs mediates gastric cancer induced angiogenesis. (**A**) Knockdown of ANG2 in ECs reduced their tube formation capacity (upper panel, magnification, 40×). Treatment of shANG2-ECs with shCtrl-AGS-EVs or with recombinant human ANG2 (huANG2) increased their tube formation capacity, whereas treatment with shANG2-AGS-EVs had no effect. Treatment with TIE2 inhibitor prior to the addition of shCtrl-AGS-EVs diminished their ability to form tubes (lower panel, magnification, 100×; scale bar represents 50 µm). (**B**) Number of tubes quantification. *** *p* < 0.001.

We used our in vitro model of ANG2 knockdown GC cells and ECs to specifically assess whether ANG2 is essential for AGS-EV-induced angiogenesis. First, we compared the ability of the ANG2 knockdown ECs (shANG2-ECs) to form capillary-like structures compared to ANG2-expressing ECs (shCtrl-ECs)**.** As depicted in Figure 8A (upper panel), knockdown of ANG2 in ECs reduced their ability to form tubes by fourfold compared to ANG2-expressing ECs (*p* = 0.0001). Next, we treated ANG2 knockdown ECs with shANG2-EVs vs. shCtrl-EVs and evaluated their ability to restore the formation of capillary-like structures. As shown in Figure 8A (lower panel), ANG2-positive EVs (shCtrl-EVs) increased the ability of the ANG2 knockdown endothelial cells to form capillary-like structures by 3.4-fold (*p* = 0.0001). Similarly, human recombinant ANG2 increased their tube formation by threefold (*p* = 0.0003), whereas treatment with TIE2 inhibitor prior to the addition of shCtrl-EVs completely diminished this effect. These results suggest that GC-EV-derived ANG2 may mediate GC-induced angiogenesis.

## 4. Discussion

In the present study, we demonstrated the proangiogenic effect of gastric cancer-derived EVs (GC-EVs). We describe, for the first time, the angiogenic protein profile of these EVs and show that their proangiogenic effect is specifically mediated through the transference of ANG2 and PI3K/Akt activation.

Growing evidence points toward the role of cancer-derived EVs in intercellular communication and cancer progression. These studies described the role of EVs as specialized messengers, covering the different stages of cancer progression, including growth and metastasis, tumor metabolism, chemoresistance, and angiogenesis [45]. While several recent studies have shown that cancer-derived EVs modulate the tumor microenvironment by enhancing angiogenesis [46], data on GC-EVs and angiogenesis are scarce. We now showed that GC-EVs are transferred into endothelial cells, thus inducing endothelial cell proliferation, migration, invasion, and tube formation both in vitro and in vivo. These data support recent reports that showed that angiogenesis was promoted by the treatment of HUVECs with GC cell-derived exosomes. Those authors demonstrated that transmission of specific miRNAs and other noncoding RNAs by GC-EVs prompts angiogenesis and gastric cancer tumor progression [47,48,49,50,51]. While these few reports focused on the role of transported RNAs, there is only a single report that described the effect of GC-EV-derived proteins on angiogenesis. In that study, Xue et al. showed that Y-box-binding protein 1 (YB-1) transferred by GC exosomes into HUVECs promotes angiogenesis by enhancing the expression of VEGF, ANG1, MMP-9, and IL-8 in endothelial cells [52].

We used a comprehensive approach to characterize angiogenic proteins in GC-EVs. Our proteomic analysis revealed the expression of various pro- and antiangiogenic factors within GC-EVs. Interestingly, we observed a high expression of proangiogenic proteins (i.e., ANG1, ANG2, VEGF, MMP1, uPAR, CXCL11, IL-6, IGF-I, and TIMP-1) compared with a relatively low expression of antiangiogenic proteins (i.e., angiostatin, endostatin, IL-4, TIMP-2, and INF-gamma). Our findings are supported by a few other studies that demonstrated the angiogenic profile of EVs from different tumor cells. Analysis of exosomes from glioblastoma cells showed that they are enriched with the proangiogenic factors VEGF, angiogenin, TGFβ, IL-8, IL-6, MMP2, MMP9, TIMP-1, TIMP-2, and CXCR4 [53,54]. Another study showed that melanoma-derived exosomes contain VEGF, IL-6, and MMP2 [55]. As for the ratio between pro- and antiangiogenic properties of tumor-derived EVs, a recent study demonstrated that exosomes from nasopharyngeal carcinoma cells contain high levels of proangiogenic proteins including CD44 isoform 5, ICAM-1, and MMP13, in contrast to low levels of the antiangiogenic protein, thrombospondin-1 [56]. Our results support existing data by showing that cancer cells reprogram their surroundings into a tumor-promoting microenvironment via the secretion of EVs [57]. In order to create their own vascular system, tumors change their microenvironment from an antiangiogenic to a proangiogenic one via a process termed “angiogenic switch” [29,58]. Our report supports the premise that EVs may be specifically involved in such a cancer-related angiogenic switch; moreover, this is the first study to demonstrate the role of GC-EVs in this process.

ANG2 was one of the most abundant angiogenic proteins in the present proteomic analysis. Significant upregulation of tissue and blood ANG2 levels has been reported in many types of cancer, including melanoma, glioblastoma, breast cancer, renal cell carcinoma, colorectal cancer, and GC [14,17,59,60], but the transference of ANG2 via tumor EVs into the microenvironment has only been reported in hepatocellular carcinoma (HCC). In their study, Xie et al. demonstrated that ANG2 existed in HCC-derived exosomes and was delivered into HUVECs via exosome endocytosis to stimulate angiogenesis [61].

The expression of ANG2 in primary and metastatic tumors has been described in breast and prostate cancers. There was a significant correlation between ANG2 expression, lymph node metastasis, tumor grade, and lymph-vascular invasion in breast cancer specimens [62], whereas ANG2 was correlated to the Gleason score and metastases in prostate cancer [63]. The expression of ANG2 has been reported in primary GC tissues [14,15], and our analysis of primary GC tissues corroborates those findings. In addition, we now show that ANG2 is also expressed in GC omental metastasis specimens. Taken together, these results imply that ANG2 may promote gastric cancer metastasis by stimulating angiogenesis in the omental metastatic niche; however, further studies are necessary to validate this preliminary finding.

The angiogenic activity of ANG2 was demonstrated through binding to TIE2 and activation of the PI3K/Akt signaling pathway [11]. In the current study, GC-EVs induced the expression of ANG2 and the downstream proteins PI3K and p-Akt in ANG2 knockdown endothelial cells. We hypothesize that ANG2 delivered by GC-EVs may be recycled by endothelial cells after internalization and excreted to bind TIE2, thus activating the PI3K/Akt signal transduction pathway. An additional support for our hypothesis was demonstrated in the tube formation assay of the ANG2 knockdown endothelial cell model. Recombinant human ANG2, as well as EVs containing ANG2, could restore tube formation of ANG2 knockdown endothelial cells. This effect was completely eliminated when the cells were treated with TIE2 inhibitor before treatment.

Antiangiogenic therapies play a role in the treatment of metastatic cancer patients. Most of these therapies target the VEGF signaling pathway and have shown efficacy in many types of cancer. However, their long-term efficacy is limited due to drug resistance. Since angiogenesis is crucial for cancer progression and metastasis, there is a clear need for novel antiangiogenic therapeutic targets. Over the past decade, the ANG–TIE signaling pathway has been the subject of much research in search of a complement to the current VEGF-based anti-angiogenic therapies. To date, several drugs targeting the ANG2/TIE2 signaling pathway are under clinical trials as adjunct cancer therapies [16]. We focused on GC microenvironment, specifically endothelial cells, as a potential target for therapy. Our data suggest that ANG2 and the ANG/TIE pathway comprise a potential target for therapy for gastric cancer patients. Moreover, we present novel findings which support the activation of this signaling pathway as being mediated by gastric cancer EVs. Thus, these EVs should be further investigated as a potential angiogenic target for therapy. To date, there are no existing anti-EV therapies in clinical practice or clinical trials which evaluate their efficacy. However, over the past years, various inhibitors of EVs have been studied, including pharmacological inhibitors of EV release, particularly inhibitors of EV trafficking and those that affect lipid metabolism [64]. Hopefully, understanding the biogenesis of EVs in the near future will unravel specific mechanisms of EV-induced angiogenesis, a key factor in the development of new gastric cancer therapy strategies. We believe that the transportation mechanism should be further investigated in the next years as a potential therapeutic approach for gastric cancer patients.

The present study harbors several limitations. Firstly, we performed immunohistochemistry for ANG2 in primary and metastatic GC human tissues and used it as a proof of principle. Thus, conclusions should be carefully made regarding the correlation between cancer differentiation and ANG2 expression. To do so, a large cohort of gastric cancer patients should be investigated. Secondly, the ANG–TIE signaling pathway is complex and affected by numerous molecules such as ANG1, VEGF, and HIF1α. These interact as agonists and antagonists to the TIE receptor. Therefore, further investigation is needed prior to the potential development of efficient anti-TIE therapies. Lastly, we used EVs which were produced from two GC cell lines. It is possible that EVs from fresh human gastric cancer tumors are affected by their native tumor microenvironment; thus, their content differs. We plan to adopt this approach to improve our biological model.

## 5. Conclusions

We demonstrated that GC-EVs promote angiogenesis by enhancing endothelial cells migration, invasion, and tube formation. Proteomic analysis of these EVs revealed high expression of a proangiogenic protein, ANG2, supporting the premise that these EVs are important mediators of tumor angiogenesis. This study provides a new mechanism via which GC cells induce angiogenesis. Such a mechanism may be used as a target for antiangiogenic therapy.

## Data Availability

The data presented in this study are available on request from the corresponding author.

## Supplementary Materials

The following supporting information can be downloaded at: https://www.mdpi.com/article/10.3390/cancers14122953/s1: Figure S1. Uncropped Western blot images corresponding to Figure 2; Figure S2. Uncropped Western blot images corresponding to Figure 7.
