# Supplementary material for: Gastric Cancer-Derived Extracellular Vesicles (EVs) Promote Angiogenesis via Angiopoietin-2
Source: Cancers (Basel). 2022 Jun 15;14(12):2953. doi: 10.3390/cancers14122953 (PMC9221039; doi:10.3390/cancers14122953)
Supplement: Supplementary file 1 [file cancers-14-02953-s001.zip › cancers-1730704-supplementary.pdf]

cancers

Article

Gastric Cancer-Derived Extracellular Vesicles (EVs) Promote Angiogenesis via Angiopoietin-2

Talya Kalfon, Shelly Loewenstein, Fabian Gerstenhaber, Stav Leibou, Hen Geller, Osnat Sher , Eran Nizri and Guy Lahat

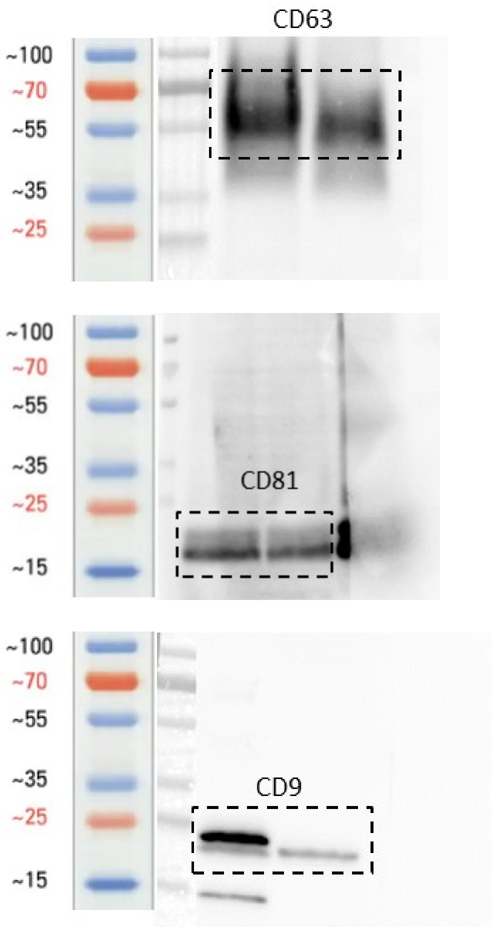

Figure S1. Uncropped western blots images corresponding to Figure 2.

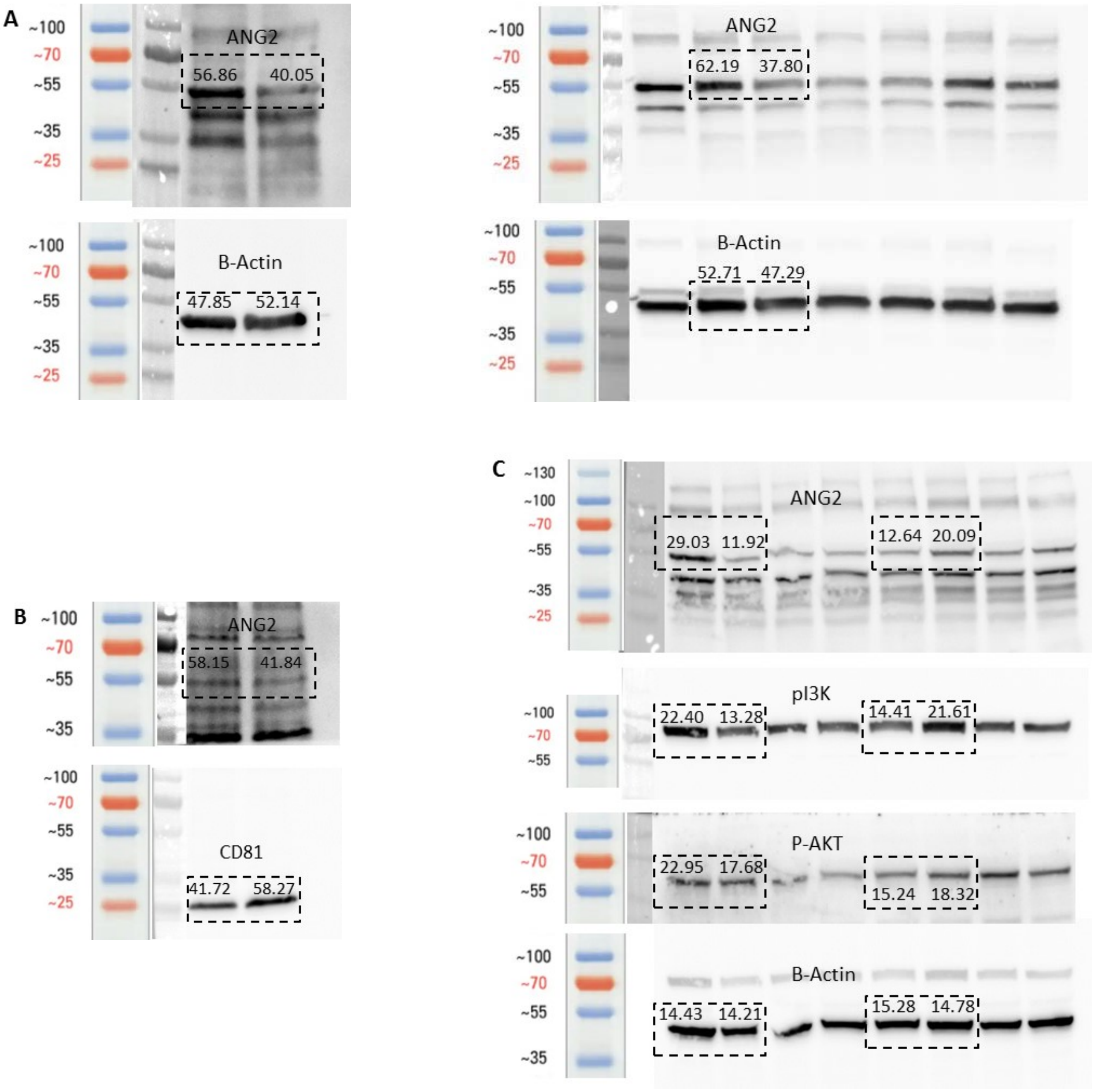

Figure S2. Uncropped western blots images corresponding to Figure 7.
